# Effects of preferred-exercise prescription compared to usual exercise prescription on outcomes for people with non-specific low back pain: a randomized controlled trial [ACTRN12608000524392]

**DOI:** 10.1186/1471-2474-10-14

**Published:** 2009-01-28

**Authors:** Susan C Slade, Jennifer L Keating

**Affiliations:** 1 Department of Physiotherapy, School of Primary Care, Faculty of Medicine, Nursing and Health Sciences, Monash University, PO Box 527, Frankston, Victoria 31990, Australia; 2 PO Box 1241, Box Hill, Victoria 3128, Australia

## Abstract

**Background:**

Non-specific chronic low back pain (NSCLBP) has become a significant problem due to high healthcare utilization, rising costs of care and perceived limitations of effectiveness of many current treatments. Systematic reviews have repeatedly concluded that, on average across participants, exercise for NSCLBP appears effective in decreasing pain and improving function. Not all people with NSCLBP benefit from exercise programs and it would assist care-providers and care-seekers if factors that impact on program effectiveness and success were identified.

**Methods and design:**

The study will be a randomised controlled trial comparing an exercise rehabilitation program informed by a participant preferences questionnaire compared to a program without this guideline for patients with chronic low back pain. A sample of 150 patients will be recruited in Melbourne, Australia through community-based healthcare clinics that provide supervised exercise rehabilitation programs for people with non-specific chronic low back pain. Clinicians will be randomly assigned to exercise preferences questionnaire or no questionnaire and participants will be allocated in a concealed manner. A qualitative focus group study of exercise instructor feedback about the exercise preferences instrument will be embedded in the research design. Two qualitative focus group studies will also be conducted for participants in the intervention and the control groups to obtain feedback about participants' experiences of the two types of exercise programs. The primary outcomes will be functional ability, pain, fear avoidance, exercise adherence.

**Discussion:**

This trial will evaluate the effectiveness of individualised exercise prescription compared to usual exercise prescription for NSCLP and, using feedback following the trial, refine the exercise preferences questionnaire.

## Background

Non-specific chronic low back pain (NSCLBP) has become a significant problem due to high healthcare utilization, rising costs of care and perceived limitations of effectiveness of many current treatments. It is a significant source of long-term disability and absence from work and a substantial burden in industrialized societies [1-9]. NSCLBP is not a diagnosis but rather a description of back pain for which a cause cannot be definitively identified and a precise patho-anatomical diagnosis cannot be given. This accounts for approximately 85% of all low back pain and does presume that specific pathologies, such as nerve root compression or tumour, have been ruled out by appropriate tests and imaging [10]. The condition manifests as a continuation of an initial episode or periodic recurrences and remissions [11,12].

Systematic reviews have repeatedly concluded that, on average across participants, exercise for NSCLBP appears effective in decreasing pain and improving function [13-20]. Many exercise programs have been designed and quite different programs appear to have similar effects. Not all people with NSCLBP benefit from exercise programs and it would assist care-providers and care-seekers if factors that impact on program effectiveness and success were identified [21,22]. Clarification of the ingredients that make exercise programs enjoyable and inviting would enable clinicians to prescribe interventions that are appropriate for the NSCLBP care-seeking population. A systematic search of the literature demonstrated that there is no available instrument that measures patient experience of exercise rehabilitation programs for NSCLBP. It is currently unknown whether patient expectations are met or what they experience during exercise programs for NSCLBP.

Following Monash University Standing Committee on Ethics in Research Involving Humans (SCERH) approval (SCERH CF07/1854 – 2007/0558), we conducted qualitative focus group research of participant experience of exercise programs for NSCLBP and factors that they perceived important for engagement and participation. The results of this study indicated that people with NSCLBP have a range of environmental and exercise design preferences that are important to consider in exercise prescription. Enablers for exercise participation included shared decision-making, effective communication, a history of exercise ability and participation, familiarity with exercise environments and the fitness culture, individualised and supervised programs, use of preferred environments, family support, variety and fun, motivation strategies and education. Barriers included time constraints, cost, boring programs, symptom aggravation, consequences of stigma, dissatisfaction with formal exercise and gym 'culture' [23-25].

A decision-aid for exercise prescription is currently not available and the results of our qualitative research have informed the development of a questionnaire that clinicians can use to determine participant exercise preferences and inform their practice of exercise prescription. This questionnaire requires testing in the clinical environment to determine its utility as a method of systematic evaluation of care-seeker needs and preferences related to exercise. The research plan is to implement the questionnaire in a range of exercise programs that are specific to NSCLBP, evaluate the effectiveness of individualised exercise prescription and evaluate feedback about the instrument.

## Methods/Design

**Figure 1:**
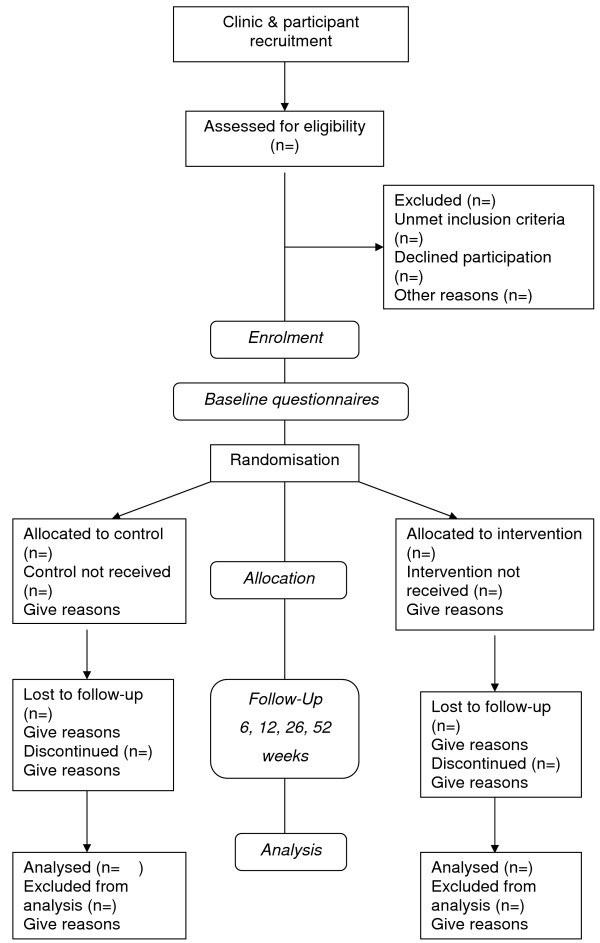
**Participant flow through the RCT (based on CONSORT Statement)**.

Monash University SCERH has granted approval for this study (CF08/2015 – 2008000990). The trial will be reported according to the recommendations of the CONSORT Statement [26,27] and the flow of participants through the study is represented in Figure 1. The quantitative study will compare an exercise rehabilitation program informed by a participant preferences questionnaire compared to a program without this guideline for patients with NSCLBP. The qualitative study will evaluate clinician and participant experience. Patients presenting for exercise prescription will be allocated to clinicians who either do or do not use information regarding patient exercise preferences.

### Quantitative study

#### • Design

The study will be a randomised controlled trial (RCT) with an intervention period of six weeks and exercise programs will be those of usual clinician practice. Outcomes will be measured at baseline and at 6, 12, 26 and 52 weeks from baseline.

#### • Controlling bias

The RCT design includes key method features that have been recognised as important in minimising bias in clinical trials: true randomisation, concealed allocation, specification of eligibility criteria, blind outcome assessment, blind analysis and intention-to-treat analysis.

#### • Setting

A sample of patients will be recruited in Melbourne, Australia through community-based healthcare clinics that provide supervised exercise rehabilitation programs for people with NSCLBP.

#### • Protocol protection

The following mechanisms will be used to ensure that the trial protocol is applied consistently: protocol manuals will be developed and all involved researchers will be trained to ensure that screening, assessment, random allocation and treatment procedures are conducted according to the protocol; a random sample of treatment sessions will be audited to check that treatment is administered as per the protocol.

### Study population and recruitment

#### • Clinics and clinicians

The study will be conducted in Melbourne metropolitan primary health care clinics that provide exercise programs for rehabilitation of musculoskeletal conditions. The contact details used will be those publicly available in the telephone directory or online at professional association websites. The clinics will be approached by telephone or email/mail advertisement seeking expressions of interest in research participation.

A phone conversation or meeting will be scheduled with the practice principal to outline the project and distribute the Explanatory statement and Clinic Consent Form. After receipt of signed Clinic Consent, information sessions will be scheduled to address clinicians and inform them of the research background and aims. They will be given an Explanatory Statement to read and keep and a Clinician Consent Form to sign and return to the researcher if they wish to participate. Reception staff in-service training will also be conducted to familiarize clinic staff with the recruitment process, paperwork completion and filing.

#### • Patients

The opportunity to participate will be offered to every eligible consecutive low back pain patient in order of contact until adequate numbers of participants are achieved. Clinic reception staff who are not aware of the research protocol or aims will approach potential participants who are patients seeking treatment for low back pain and use a screening checklist to determine eligibility and interest. They will record the number of patients invited, the number who decline, ineligible patients and reasons [see Additional file 1].

### Concealment

#### • Patients and receptionists

If patients are eligible and interested, reception staff will give them the Explanatory Statement to read and keep and a Consent Form that they will sign and return to the receptionist for filing. Following signed consent participants will complete coded outcome measures and exercise preference questionnaires and return them sealed to reception staff for storage in a secure box for retrieval by the researcher. The completed exercise preferences questionnaire will be put in a sealed envelope, marked EQ, and forwarded to the treating clinician. The envelope will only be opened by the clinicians allocated to the questionnaire group. Reception staff and patients will not know which practitioners have been allocated to use the completed exercise preference questionnaire to guide exercise prescription or to the control group.

### Randomization

#### • Clinicians

Clinicians who have consented to participate will be randomly allocated, by computer-generated codes (code: exercise questionnaire group (EQ); no exercise questionnaire group (NQ)) in sealed opaque envelopes, to one of two groups with stratification to allow equal representation of qualifications. These codes will be generated by an independent researcher who has no contact with trial participants. Group 1 clinicians allocated to use the exercise preferences questionnaire will use the responses to the exercise preferences questionnaire to inform their exercise prescription. Group 2 clinicians do not use the exercise preferences questionnaire and will return the unopened envelope to a secure storage box in the reception area. These clinicians will prescribe exercise according to their usual practice.

The two groups of clinicians will not disclose their group allocation or any details or findings of the questionnaire. A code-breaker master document will be kept in a locked location at the university and cannot be viewed by reception staff or clinicians.

### Eligibility assessment

#### • Clinicians

Health care practitioners who hold recognized qualifications in physiotherapy, exercise physiology (masters degree), chiropractic, osteopathy or certificate 4 training, who are registered to practice in Victoria and who prescribe exercises are eligible to participate.

#### • Patients

At the initial consultation clinicians will use a screening checklist to confirm eligibility. If participants meet the inclusion criteria the participant and their data will be included in the study. Eligibility criteria are reported in Table 1.

**Table 1:** RCT inclusion and exclusion criteria

**Inclusion criteria**
• over 18 years of age
• non-specific chronic low back pain: pain in the area extending from twelfth rib to buttock fold +/- leg for > 8 weeks
• receiving exercise prescription
• ability to speak, read and write English

**Exclusion criteria**

• severe cardiovascular or metabolic disease
• inflammatory disease
• spinal tumour or fracture
• nerve root compression/compromise
• spinal cord irritation/cauda equina signs
• osteoporosis.
• Pregnancy

### Blinding

#### • Clinicians

The clinicians will be asked to agree not to disclose group allocation or questionnaire details to patients, colleagues or researchers.

#### • Patients

The plain language Explanatory Statement and Patient Consent Form will inform participants that they have an equal chance of receiving one of two acceptable exercise prescription methods.

#### • Assessor

A blinded examiner will perform all outcome assessments. Participants will be asked to refrain from discussing their treatment with the outcome assessor. At trial completion, participants will be asked to nominate whether they had been in the experimental or control group. The data manager and statistician will be unaware of treatment allocation until completion of analyses.

### Sample Size

A total of 120 patients (60 questionnaire group, 60 no questionnaire group) will be required for 80% power to detect a 5 point reduction on 100 point measure of function with 95% confidence using a two-tail test of difference between group means when raw scores have an estimated standard deviation of 10/100 points). We will allow for a 10% loss to follow-up and aim to recruit 75 participants per group. This will enable collection of data for at least 85% of enrolled participants.

### Outcome measures

Socio-demographic data including age, back pain history and occupation status, will be collected at baseline. The following outcomes will be assessed at baseline, at completion of the 6 week intervention and at 12, 26 and 52 weeks: Functional ability: Oswestry Disability Index (ODI) [28], Pain: Tagged Numeric Pain Scale (NPS) [29], Fear avoidance: Fear Avoidance Beliefs Questionnaire (FABQ) [30]. The Exercise Preferences Questionnaire will be completed at baseline only and the Exercise Diary will be completed after each exercise session during the intervention and follow-up period. Time to complete the Exercise Preferences Questionnaire will be evaluated by asking the participant to note the time taken on page two of this form. Follow-up reminders will be given by phone or SMS and completed outcome measures returned by pre-paid envelope or SMS.

### Adverse effects or events

No adverse events are anticipated but will be documented by type, length of time and frequency should they occur.

### Data Analysis

Analysis will be by intention to treat. As scores are expected to improve across time, missing data will be replaced by the last score carried forward. If mean scores deteriorate across time, missing data will be replaced by the last score minus the reduction in scores anticipated using trend analysis of previous scores. Demographic characteristics and baseline data will be summarised by descriptive statistics. Distributions of all continuous data will be assessed for normality. Between-group comparisons of normally distributed data will be performed with independent t tests. Non-normally distributed data will be compared with the Mann Whitney U statistic. Relative risks and their 95% confidence intervals will be used to report group differences in proportions for similar outcomes. Continuous variables will be assessed for relationships between baseline scores and outcome. If a significant relationship is found, analysis of covariance (ANCOVA) will be conducted to test the effect of the intervention when controlling for the confounding effects of baseline scores on outcome. Effect sizes with 95% confidence intervals will be calculated for pain and function measures.

### Qualitative studies

#### • Design overview

**Figure 2:**
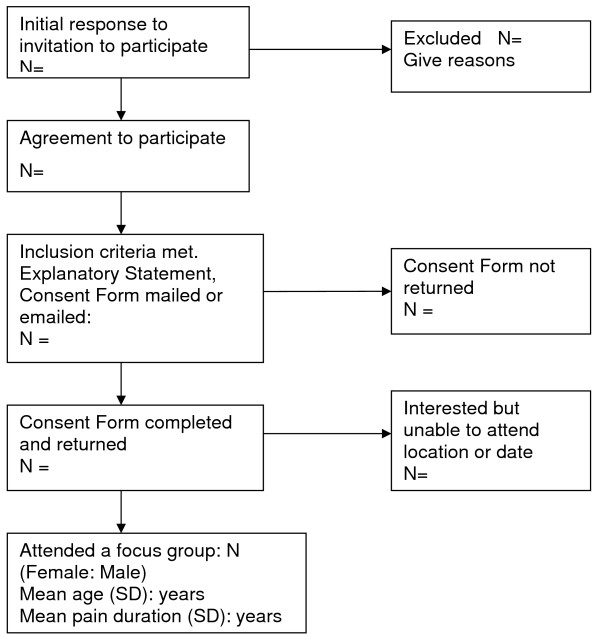
**Progress through the stages of selection for focus group studies**.

Two qualitative studies will be embedded within the RCT: (1) exercise instructor (clinician) feedback about the exercise preferences instrument (2) participant feedback about their exercise experience. The focus group research methods will follow the recommendations of Strauss and Corbin (1998) and Krueger and Casey (2000) [31,32]. Both of these studies will be conducted within one month of RCT completion. The focus groups will be 2.5 hours duration and conducted by an experienced facilitator with a pre-determined set of questions. The sessions will be audio-taped, minuted and transcribed verbatim for independent analysis of emergent themes. The flow of participants through the studies is illustrated in Figure 2.

#### • Controlling bias

Rigour of the qualitative research design will be enhanced by testing against four constructs: Credibility: independent data review, coding and theme development by at least 2 reviewers with several rounds of discussion. Transferability: *a priori *recruitment methods that reduce bias and participant heterogeneity. Dependability: accurate verbatim transcription of audio tapes and comparisons of transcript and tape content. Confirmability: consistent emergent themes from subsequent data.

### Study population and recruitment

#### • Clinicians

Clinicians who have participated in the RCT, who were allocated to the intervention (exercise preferences questionnaire group) and who have given consent to participate in focus group research will be invited to participate.

#### • Patients

Following completion of the 6 week intervention outcome assessments, participants in the RCT will be invited to participate in focus group studies to elicit feedback about their experiences of the program.

### Data collection

Focus groups will be convened at Monash University – Peninsula campus, or another convenient location. People will only volunteer information if they wish to do so. An experienced facilitator will use a discussion outline to guide the conversation and participants will be encouraged to talk freely and spontaneously. The focus group sessions will be audio-taped and a note-taker will record field notes. Immediately following each focus group the facilitator and note-taker will meet and complete a debrief form.

#### • Data storage and confidentiality

Subjects will be assigned a code number at the first contact and a pseudonym when tapes are transcribed. This code number will be the only identifying mark on all subsequent data.

#### • Data analysis

Grounded Theory will be applied in the analysis of the shared and divergent experiences of clinicians who used the questionnaire for exercise prescription and patients who participated in the exercise programs. The method is based on identification of coded themes in recorded data and identification of relationships between themes.

## Discussion

We have presented the rationale and design of a randomized controlled trial, with embedded qualitative studies, to investigate and evaluate preferred-exercise prescription compared to usual exercise prescription on outcomes for people with non-specific chronic low back pain. The results of this research will be presented as soon as they are available.

## Abbreviations

The following abbreviations have been used in the manuscript: NSCLBP: non-specific chronic low back pain; SCERH: Standing Committee on Ethics in Research Involving Humans; and RCT: randomised controlled trial

## Competing interests

The authors declare that they have no competing interests.

## Authors' contributions

SCS and JLK designed the research project. SCS will act as trial coordinator. SCS and JLK will read and approve final manuscripts.

## Pre-publication history

The pre-publication history for this paper can be accessed here:



## Supplementary Material

Additional File 1 **RCT recruitment checklist.** Form for use by reception staff to recruit participants into the study. Click here for file

## References

[B1] Andersson GB (1999). Epidemiological features of chronic low back pain. The Lancet.

[B2] Cassidy JD, Carroll LJ, Cote P (1998). The Saskatchewan Health and Back Pain Survey. Spine.

[B3] Croft PR, Dunn KM, Raspe H (2006). Course and prognosis of back pain in primary care: the epidemiological perspective. Pain.

[B4] LeBoeuf-Yde C, Klougart N, Lauritzen T (1996). How common is Low Back Pain in the Nordic Population. Spine.

[B5] Thomas E, Silman AJ, Croft PR, Papageorgiou AC, Jayson MIV, Macfarlane GJ (1999). Predicting who develops low back pain in primary care: a prospective study. BMJ.

[B6] van Tulder MW, Koes BW, Bouter L (1995). A cost-of-illness study of back pain in the Netherlands. Pain.

[B7] Walker BF, Muller R, Grant WD (2003). Low back pain in Australian adults: The economic burden. Asia Pac J Public Health.

[B8] Walker BF, Muller R, Grant WD (2004). Low back pain in Australian adults: health provider utilization and care seeking. J Manip Physiol Ther.

[B9] Walker BF, Muller R, Grant WD (2004). Low back pain in Australian adults: prevalence and associated disability. J Manip Physiol Ther.

[B10] Maher CG, Latimer J, Refshauge K (1999). Prescription of activity for low back pain: what works?. Aust J Physiother.

[B11] Hestbaeck L, LeBoeuf-Yde C, Manniche C (2003). Low back pain: what is the long term course? A review of studies of general patient populations. Eur Spine J.

[B12] Pengel LH, Herbert RD, Maher CG, Refshauge KM (2003). Acute low back pain: systematic review of its prognosis. BM J.

[B13] Clare HA, Adams R, Maher CG (2004). A systematic review of the efficacy of McKenzie therapy for spinal pain. Aust J Physiother.

[B14] Ferreira PH, Ferreira ML, Maher CG, Herbert RD, Refshauge K (2006). Specific stabilisation exercise for spinal and pelvic pain: a systematic review. Aust J Physiother.

[B15] Hayden JA, van Tulder MW, Malmivaara A, Koes BW (2006). Exercise therapy for treatment of non-specific low back pain. The Cochrane Library.

[B16] Hayden JA, van Tulder MW, Malmivaara A, Koes BW (2005). Meta-analysis: Exercise Therapy for treatment of non-specific low back pain. Ann Intern Med.

[B17] Hayden JA, van Tulder MW, Tomlinson G (2005). Systematic review strategies for using exercise therapy to improve outcomes for chronic low back pain. Ann Intern Med.

[B18] Machado LA, de Souza MS, Ferreira PH, Ferreira ML (2006). The McKenzie method for low back pain: a systematic review. Spine.

[B19] Schonstein E, Kenny DT, Keating J, Koes B (2004). Work conditioning, work hardening and functional restoration for workers with back and neck pain. The Cochrane Library.

[B20] van Tulder MW, Malmivaara A, Esmail R, Koes BW (2000). Exercise therapy for low back pain: a systematic review within the framework of the Cochrane Collaboration Back Review Group. Spine.

[B21] Slade SC, Keating JL (2006). Trunk strengthening exercise for chronic low back pain: a systematic review. J Manip Physiol Ther.

[B22] Slade SC, Keating JL (2007). Unloaded movement facilitation exercise compared to no exercise or alternative therapy on outcomes for people with non-specific chronic low back pain: a systematic review. J Manip Physiol Ther.

[B23] Slade SC, Molloy E, Keating JL "Listen to me. Tell me": a qualitative study of partnership in care for people with non-specific chronic low back pain. Clinical Rehabilitation.

[B24] Slade SC, Molloy E, Keating JL (2009). Stigma experienced by people with non-specific chronic low back pain: a qualitative study. Pain Medicine.

[B25] Slade SC, Molloy E, Keating JL Enablers and barriers to participant engagement in exercise programs for non-specific chronic low back pain: a qualitative study. JBJS (British volume).

[B26] Moher D, Schulz KF, Altman DG (2001). The CONSORT statement: revised recommendations for improving the quality of reports of parallel-group randomized trials. Ann Intern Med.

[B27] Altman DG, Schulz KF, Moher D, Egger M, Davidoff F, Elbourne D, Gøtzsche PC, Lang T (2001). The revised CONSORT statement for reporting randomized trials: explanation and elaboration. Ann Intern Med.

[B28] Davidson M, Keating JL (2002). A comparison of five low back disability questionnaires: reliability and responsiveness. Phys Ther.

[B29] Farrar JT, Young JP, LaMoreaux L, Werth JL, Poole RM (2001). Clinical importance of changes in chronic pain intensity measured on an 11-point numerical pain rating scale. Pain.

[B30] Waddell G, Newton M, Henderson I, Somerville D, Main CJ (1993). A Fear-Avoidance Beliefs Questionnaire (FABQ) and the role of fear-avoidance beliefs in chronic low back pain and disability. Pain.

[B31] Krueger RA, Casey M (2000). Focus Groups: a practical guide for applied research.

[B32] Strauss A, Corbin J (2000). Basics of Qualitative Research: Techniques and procedures for developing grounded theory.

