# Supplementary material for: Effects of preferred-exercise prescription compared to usual exercise prescription on outcomes for people with non-specific low back pain: a randomized controlled trial [ACTRN12608000524392]
Source: BMC Musculoskelet Disord. 2009 Jan 28;10:14. doi: 10.1186/1471-2474-10-14 (PMC2642754; doi:10.1186/1471-2474-10-14)
Supplement: Additional File 1 — RCT recruitment checklist. Form for use by reception staff to recruit participants into the study. [file 1471-2474-10-14-S1.pdf]

MONASH University
Medicine, Nursing and Health Sciences

Effect of preferred exercise prescription for people with non-specific low back pain

Reception staff: Checklist for invitation to participate

1. Have you had low back pain for longer than 8 weeks? ☐yes ☐no

2. Do you understand that we will not interfere with treatment choice?

   ☐yes ☐no

3. Are you interested to participate in a study of the best practice in

   exercise prescription for back pain? ☐yes ☐no

4. Do you understand that you are welcome not to participate?

   ☐yes ☐no

5. Non-participation will not affect your treatment in any way

   ☐yes ☐no

Proceed to Explanatory Statement and Consent Forms ☐yes ☐no

Receptionist Name: ………………………………………………………………

Receptionist Signature: ……………………….**Date:** …… /…..… / ……........
